# Research on Low-Cycle Fatigue Engineered Hybrid Sandwich Ski Construction

**DOI:** 10.3390/polym14112278

**Published:** 2022-06-03

**Authors:** Tomáš Božák, Miroslav Müller, Viktor Kolář, Martin Tichý, Jaroslava Svobodová, Štefan Michna

**Affiliations:** 1 Department of Material Science and Manufacturing Technology, Faculty of Engineering, Czech University of Life Sciences Prague, Kamycka 129, Suchdol, 165 00 Prague, Czech Republic; bozak@tf.czu.cz (T.B.); muller@tf.czu.cz (M.M.); martintichy@tf.czu.cz (M.T.); 2 Faculty of Mechanical Engineering, University of Jan Evangelista in Ústí nad Labem (UJEP), Pasteurova 3334/7, 400 01 Usti nad Labem, Czech Republic; jaroslava.svobodova@ujep.cz (J.S.); stefan.michna@ujep.cz (Š.M.)

**Keywords:** hybrid construction, delamination, testing methodology, bonding technology, low cycle fatigue, SEM

## Abstract

This research is aimed at evaluating the effect of low-cycle fatigue on a newly designed hybrid sandwich ski structure to determine the changes that may occur due to cyclic loading and thus affect its use. This is primarily concerned with the fatigue behavior of the tested ski over different time intervals simulating its seasonal use and its effect on the mechanical properties of the ski, i.e., the durability and integrity of the individual layers of the sandwich ski structure. The ski was subjected to 70,000 deflections by moving the crossbar by 60 mm according to the ski deflection calculation in the arch. The results of the cyclic tests of the engineered ski design showed no significant changes in the ski during loading. The average force required to achieve deflection in the first 10,000 cycles was 514.0 ± 4.2 N. Thereafter, a secondary hardening of the structure occurred during relaxation and the force required increased slightly to 543.6 ± 1.7 N. The required force fluctuated slightly during the measurements and in the last series the value was 540.4 ± 0.8 N. Low-cycle fatigue did not have a significant effect on the mechanical properties of the ski; there was no change in shape or visual delamination of the individual layers of the structure. From the cross-section, local delamination was demonstrated by image analysis, especially between the Wood core and the composite layers E-Glass biaxial and Carbon triaxial.

## 1. Introduction

Skis are an important segment of manufacturing companies involved in the production of sports equipment. Skis represent a complex set of technological operations in their manufacture affecting their use. The construction of skis is based on a composite hybrid sandwich construction using a variety of materials, with the use of bonding technology. Each layer performs a predetermined function in the sandwich construction of the ski. The strength in the sandwich construction is created by composite reinforcements that are bonded [1]. One of the advantages of making skis using sandwich construction is the possibility of omitting the heavy, tough core of the ski. The strength in the sandwich construction is made up of composite reinforcements [2]. Conventional manufacturers tend to use glass reinforcements in their production. These reinforcements are well bonded. For this reason, they are widely used, but they lose their properties during their service life [1]. Laminates created using glass, carbon or aramid fibers using polymer matrices are used for the outer layers of the sandwich material. For example, sheet metal is used as an additional material. Sheets are popular because of their low cost and good mechanical properties, but their disadvantage is that they increase the weight of the resulting sandwich material [3,4,5,6]. Natural or synthetic material can be used as the core material. Among natural materials, wood is widely used. These are mainly soft and flexible woods. Of the synthetic materials, polymer foams are often used. The shape of the core often mimics the honeycomb structure. Such a core can be made of, for example, metal, laminate or polymers. The honeycomb core is often used, for example, in load-bearing structures. For materials created using a sandwich construction, the resulting properties are determined, i.e., density, strength, elasticity, etc. [7,8].

Low cycle fatigue is used to determine the mechanical properties of a specific sample [3,4,9,10]. Its principle is to expose the material to a certain tension, which is cyclically repeated. At low cyclic fatigue, destruction of the sample should not occur within 10 thousand repetitions [11,12,13]. It is commonly reported that the strength and service life of products is reduced at low stress values during cyclic fatigue of materials, especially when polymer-based materials are used [3,6]. Research indicates that cyclic fatigue causes an irreversible change before the maximum strength of materials is reached [14]. While moving on skis, the skier moves mainly by rhythmic forward and backward movements of the legs [15]. Rhythmic movements occur in cycles that repeat. The ski structure tends to be subjected to low cyclic fatigue during use, which can have consequences for the integrity of the individual layers and changes in mechanical properties and shape [16].

When the mechanical properties of a sandwich ski structure are tested, it is preferable to use fatigue tests rather than static tests. Cyclic tests simulate the practical application in terms of long-term use [6]. The time aspect is also a very important factor that can influence the resulting properties. For example, the cyclic load test corresponds exactly to the real load on the ski structure during use. A cyclic load test, simulating real conditions, can determine the properties and service life of a given sandwich structure [3,10,17]. Cyclic loading has a degrading effect on the life of bonded joints in the structure, even when reinforced with different filler, reinforcing phases [17,18,19]. Cyclic loading of the bonded structure can degrade the mechanical properties and can lead to delamination of the individual layers in the structure and thus to destruction [3,4,17]. The layout of the ski is based on a hybrid sandwich laminated construction. The stress transfer between the individual layers of the hybrid sandwich construction is a very important factor in increasing the resistance to fatigue behavior [10,14]. The results from fatigue testing of products and test specimens are of high relevance and utility for practical applications, as they provide technological recommendations for the use of the materials [20].

Ski manufacturers currently focus on field testing. These tests offer valuable information, but are also limited by factors such as changing environmental conditions and the associated test parameters [21].

This article presents research that continues the experimental development that deals with the design, construction and manufacture of a prototype skialp ski. In the design and manufacture of this prototype, modern shapes and materials were used to achieve the best possible skialp and downhill skiing characteristics. The aim was to create a ski light enough to easily handle long uphill climbs, but strong enough for everyday skiing on the slopes. The research is focused on the evaluation of the effect of low cycle fatigue on a newly designed hybrid sandwich ski structure produced and developed in collaboration with Bear skis and Forest skis to determine the changes that may occur due to cyclic loading and thus affect its use, i.e., the lifetime and integrity of the individual layers of the sandwich ski structure. This is primarily concerned with the fatigue behavior of the tested ski over different time intervals simulating its seasonal use, i.e., according to time parameters based on the manufacturer’s requirements. This research will have an impact on determining the possible limits of the practical use of the product during the season.

## 2. Materials and Methods

### 2.1. Materials

**Figure 1:**
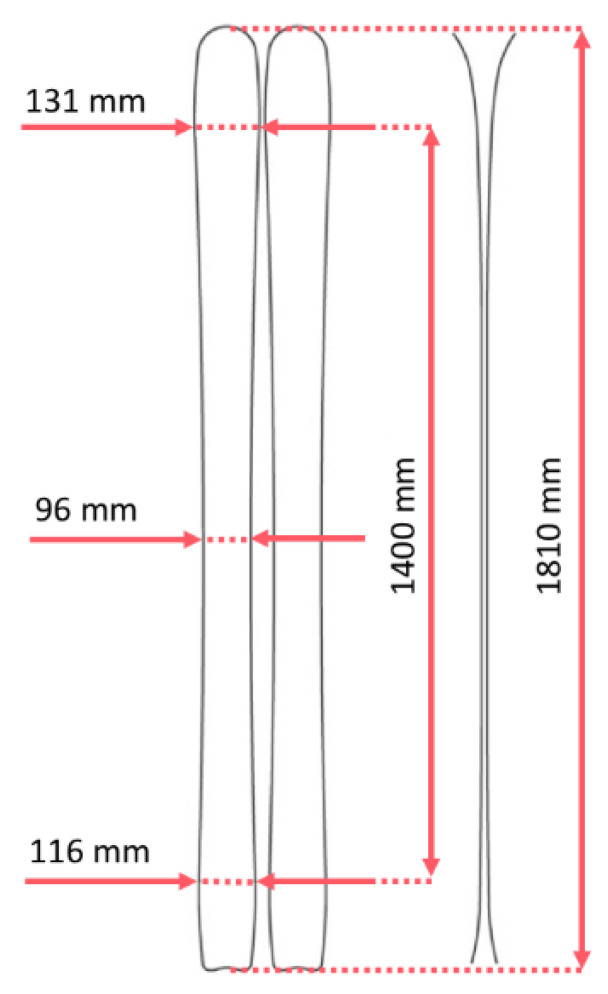
Ski parameters of newly engineered and tested skis.

A multi-purpose ski design was created for skialp use, i.e., light enough to easily handle long uphill climbs, but strong enough for everyday downhill skiing. For this reason, new technologies such as rocker were used in the design of the ski to make it more maneuverable and easier to initiate a turn. For comparison, lightweight skialp skis from traditional manufacturers Fischer Hannibal 96 [22] (Fischer Sports GmbH, Ried im Innkreis, Austria) weigh the same, at 1450 g per ski at 183 cm length, and downhill skis e.g., Armada Tracer 98 [23] (Armada Skis, Park City, UT, USA) weigh 1625 g per ski at 180 cm length. The ski was created for the average adult male. Its parameters are shown in Table 1 and Figure 1. The length was set to 181 cm and a center width of 96 mm was chosen for use off the piste. The radius of the ski was 21 m for easy handling and use on the piste (Figure 1).

**Figure 2:**
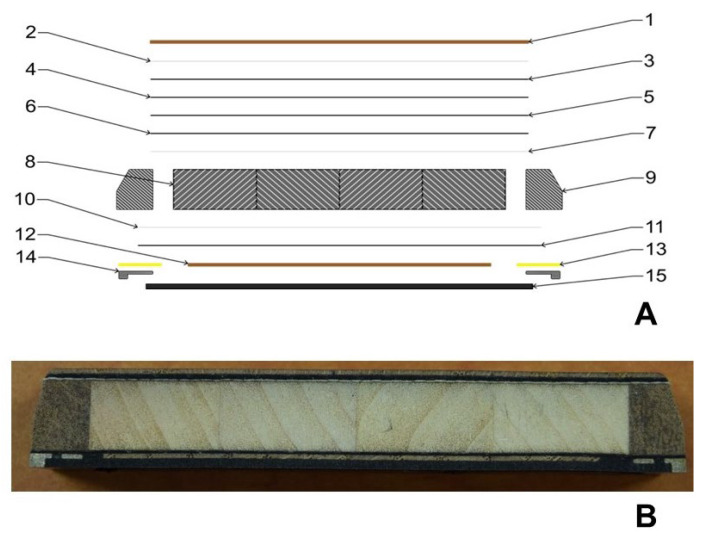
Hybrid sandwich structure of the tested ski: (**A**): Composition and relative arrangement of materials in the ski structure, (**B**): cross section of the tested ski.

Figure 2 shows the hybrid sandwich structure of the tested skis.

Figure 2A shows the different semi-finished products and their mutual arrangement in the ski structure. Figure 2B shows a cross-section of the tested ski. The materials of which the ski is made are specified in Figure 2A and Table 2.

Figure 2 presents the relative arrangement of the sandwich hybrid structure of the manufactured and tested skis, with the material composition evident from Table 2. A poplar core was chosen to lighten the ski, with carbon and glass reinforcement adding strength. Graphite was chosen for the base and hardened steel for the edge material. Carbon triaxial reinforcement together with glass biaxial reinforcement was prepared for the bottom layer of reinforcement under the core. The core is made of poplar prisms and reinforced with bangkirai wood sidewalls. The top reinforcement as well as the bottom layer consisted of carbon triaxial fiber together with glass biaxial. In addition, one layer of biaxial glass fiber and three layers of different lengths of carbon unidirectional fiber were added at the top. These layers are intended to provide sufficient strength to the structure. A thin oak veneer was chosen as the top layer.

### 2.2. Methods

The skis were produced in a pressing machine, which consisted of a frame, upper mold, lower mold, pressure distribution mat, aluminum plates, pressure system and pressing area. The production of the skis began with the preparation of all the necessary components to assemble the ski. It was necessary to cut the base to the exact shape and glue the edges to it. For the core, wood without knots and defects was selected. The core was glued together from cut squares and cut to the exact shape. The sidewalls were then bonded to the core and the entire blank was shaped to the exact form. The next step was to cut the reinforcements and the topsheet to the required size. All components had to be degreased and prepared for bonding before pressing. The individual layers of the ski were laid on the aluminum sheet one by one. It started from the bottommost to the topmost, i.e., from the base to the top layer. The individual materials had to be lubricated and saturated with epoxy resin. The semi-finished product was covered with the top sheet, placed in the pressing compartment and pressed against the bottom mold by a pressure system. After removal from the press, the product was cut with a band saw and ground to the exact shape according to the edges. The sides were beveled to the required angle and the surface finish of the face parts was carried out.

**Figure 3:**
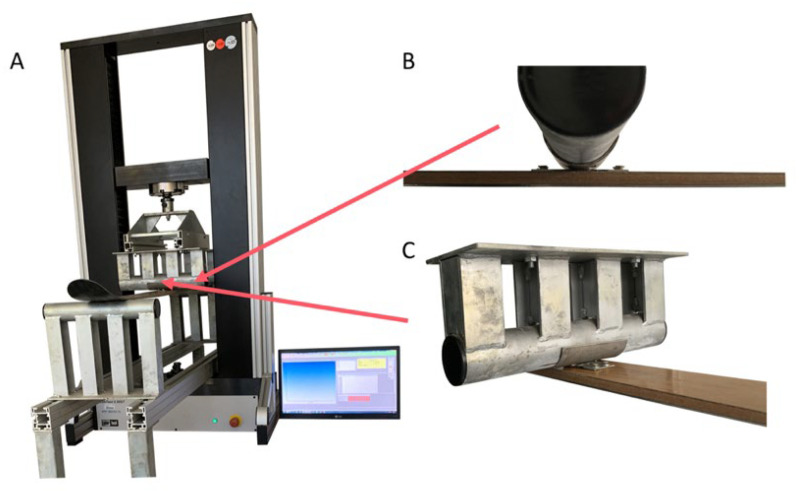
Modification of the Labortech test machine with the developed jig for testing in bending: (**A**): Test rig with special jig and ski placement, (**B**,**C**): Detailed view of jig and ski attachment.

The fatigue loading of the newly manufactured structure of the tested ski was tested on the universal testing device LabTest 5.50ST (LABORTECH s.r.o., Opava, Czech Republic) with the measuring unit AST KAF 50 kN (LABORTECH s.r.o., Opava, Czech Republic) and with the software Test and Motion (LABORTECH s.r.o., Opava, Czech Republic), enabling low-cycle fatigue testing of the ski structure in four-point bending. In order to carry out the bending test of the skis, it was necessary to use special jigs for clamping the skis to the bending stresses in the test rig, which were developed and manufactured within the research at the Department of Materials and Engineering Technology, TF, ČZU in Prague, see Figure 3.

These jigs consisted of a support bed on which different spacing and positioning of the supports could be set (Figure 3A–C). Figure 3A shows the overall arrangement during cyclic bending testing of the manufactured prototype ski. Figure 3B shows the part of the jig that exerts compression on the tested ski. Figure 3C shows the lower part of the pad between the loading mandrel and the tested ski simulating the binding attachment in a practical application.

**Figure 4:**
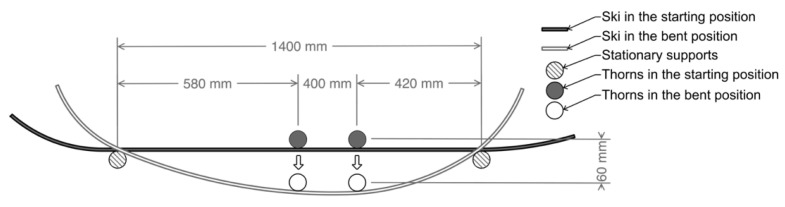
View at the ski in the testing position.

In our case, the spacing of the supports was set to 1400 mm—see Figure 4—i.e., the length of the effective edge of the ski.

**Figure 5:**
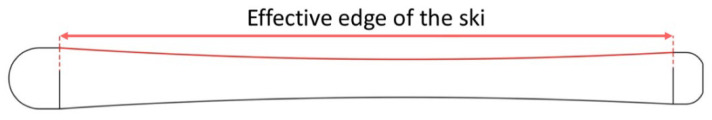
Effective edge of the ski.

An example of the effective ski edge can be found in Figure 5.

The other parts were fixed on the movable crossbar. These parts consisted of two thorns, which were intended to simulate pressure into the ski at the same points as would be the case for a skier. Mounts were attached to the ski at the mounting points to simulate contact surfaces as would be the case with a ski binding. This fixture fit precisely into the thorns on the moving crossbar, thus achieving the attachment of the ski to the machine (Figure 3B,C).

Stress and deformation data were recorded throughout the testing process, i.e., exposure to cyclic loading. Experiments on the test object, which was in the form of the final product of the newly developed ski, were carried out under controlled loading conditions at laboratory ambient temperature. Gang Tao and Zihui Xia [24] state that proper recording of the fatigue test procedure, i.e., recording especially the speed and loading progress, will have a significant effect on the fatigue behavior of the tested material. For this reason, it is very important to obtain the strain and stress data during the fatigue process [24].

The average skier tilts the ski in a carving arc, most often to 70° to the base. This value was increased by 10% to 77° for our calculations due to the slight over-dimensioning. Based on this angle and the 13.5 mm carving depth of the test sample, the test rig crossbar offset was calculated to be 60 mm using Formula (1):

X = 13.5/ctg 77°(1)

**Figure 6:**
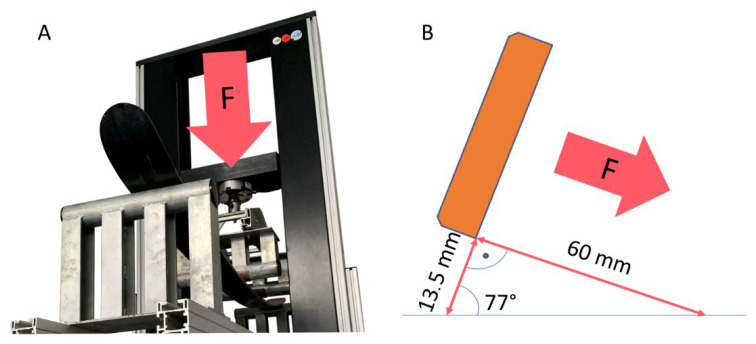
Testing: (**A**): Ski deflection in the test rig, (**B**): Ski deflection in the curve.

The modification of the test rig with the developed bending test jig and the schematic representation of the deflection of the tested ski can be seen in Figure 6.

This established the main measurement parameter. The number of deflections of the ski was determined as an additional parameter. This was set to a value of 10,000 cycles. The loading was divided into successive partial fatigue tests simulating repetitive use of the ski during the season, i.e., not in one interval. This was achieved after a total of ten partial fatigue measurements. Each sub-section consisted of 1000 deflections per 60 mm. When the extreme value was reached, the machine was stopped for 0.5 s to simulate the curves. At these extreme positions of maximum deflection, the force was read by the software (Figure 6A). This low-cycle fatigue process was repeated 10×. The subparts were measured at an interval of two weeks, with each day devoted to 1000 cycles. The test speed of the crossbar was set at 1000 mm × min^−1^. This was the maximum speed that was compatible with the AST KAF 50 kHz transducer (LABORTECH s.r.o., Opava, Czech Republic), which enabled the recording of measurement data. It is also based on proven methodology in the field of sandwich panel research. The initial preload was set to 20 N. This load was applied to observe the elastic behavior of the sample.

After the first 10,000 cycles of measurements, the ski was left to relax. This relaxation period was established to 6 months. The time was determined by the length of the interval between two skiing seasons. After the materials in the ski structure had been relaxed for a sufficient period, the mechanical properties of the ski were tested again. The chosen testing methodology continued with the same deflection of the ski as in the first 10,000 cycles. The total number of additional ski deflections was set to 60,000. The measurements were further divided into series of 10,000 cycles. This was achieved after a total of ten partial fatigue measurements. Each sub-section consisted of 1000 deflections to a value of 60 mm, as was the case in the first part of the measurement. The 10,000 cycles were measured successively over two weeks and then the ski was left to relax for one month. After the relaxation period, the measurements were repeated the same way until the required number of deflections was reached, i.e., a total of 70,000 cycles.

To detect possible delamination after fatigue tests, cuts have been made using abrasive waterjet technology, which eliminated the formation of possible delamination due to material cutting. The optimization of the cutting process was first tested on a different ski. The surface of the specimens was then prepared in the metallography laboratory. The stereoscopic microscope Olympus SZX10 (Olympus Czech Group, s.r.o., Prague, Czech Republic) and the scanning electron microscope Tescan VEGA 3 (Tescan Brno s.r.o., Brno, Czech Republic) were used to investigate the sectioning of the hybrid sandwich ski structure. The width of delamination was measured using the Gwyddion program (version 2.49, David Nečas and Petr Klapetek, Brno University of Technology, Brno, Czech Republic). The microscopic samples were coated with gold using Quorum Q150R ES (Tescan Brno s.r.o., Brno, Czech Republic). The cross-section of a hybrid sandwich ski structure after the end of long-term low-cycle fatigue was tested by SEM analysis using 5 micrometers of gold sputtered with the equipment Quorum Q150R ES-Sputtering Deposition Rate. The parameters of the SEM images can be seen from the bottom caption of the images at HV 5 kV using an Oxford SE detector.

## 3. Results and Discussion

The results are presented on a newly developed ski that was subjected to long-term fatigue loading in a four-point bend on a modified Labortech test rig equipped with the developed bend test fixture. The required quantities were recorded, and the behavior and visual condition of the specimen were observed during the test. The results of the investigation are presented in Table 3.

The average value of the force in the first part of the measurements in each cycle from 1 to 10,000 in order to achieve the required deflection of the test specimen varied only minimally and had a fluctuating character (Table 3). Its average was 514.0 ± 4.2 N. According to the standard deviation not exceeding 4.2 N, it can be seen that the measured values in each cycle from 1 to 10,000 varied only minimally. During the first series of measurements, the ski was only in elastic deformation. This behavior is confirmed by the values of the force of the preload, where the force of the ski was measured at 0 mm deflection after the load force was relieved. The difference in force from the initial value of 20 N was evaluated. The decrease in preload from the setpoint of 20 N was 6.2 ± 0.9 N lower at 10,000 cycles.

After six months of relaxation of the materials in the ski structure, they were secondarily hardened (Table 3). The average value of the force at the extreme point of deflection of each cycle from 10,001 to 20,000 was 543.6 ± 1.7 N. This average value is 29.6 N higher than the average value of the first 10,000 cycles before relaxation. The standard deviation of the measurements after relaxation decreased by 2.5 N compared to before relaxation. The resultant force required to achieve deflection varied slightly from 10,001 to 70,000 cycles. The force of the preload also showed a fluctuating character, with the force for deflection decreasing at 0 mm as the force for deflection increased to 60 mm. The greatest decrease was 12.2 ± 0.9 N at 20,000 cycles after relaxation for 6 months. As the test progressed, the force of the preload had an increasing pattern and approached the preset preload value of 20 N at 0 mm deflection between cycles 20,001 and 70,000. No permanent deformation was observed during the measurements.

**Figure 7:**
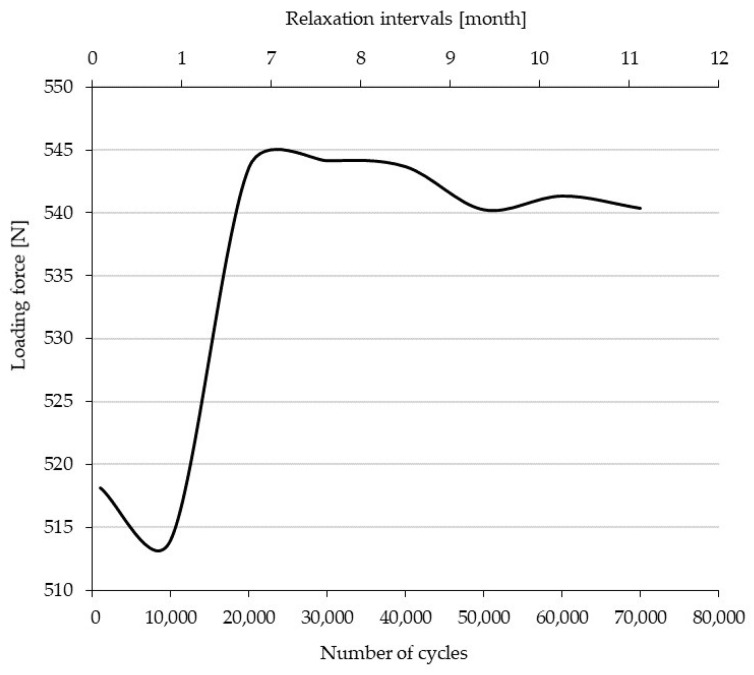
Loading force versus relaxation time in 60 mm ski bending in the interval 0–70,000 cycles.

The trend of the effect of cyclic loading on the load force of the composite hybrid ski structure can be seen in Figure 7.

The resulting load force values were always recorded when a constant deflection of the specimen of 60 mm was reached (Figure 7). The initial value of the graph is 518.1 ± 5.4 N at 1000 cycles. Subsequently, a slight decrease in loading force to 514.0 ± 4.2 N at 10,000 cycles is evident. The test specimen is set up in the test rig. Subsequently, there is a sharp increase in the loading force to 543.6 ± 1.7 N at 20,000 cycles. This increase is due to the self-reinforcing process, i.e., the carbon triaxial fibers are stretched together with the glass biaxial fibers in the matrix at the interfacial interface in the loading direction. This state of reinforcement is maintained for up to 40,000 cycles. Thereafter, there is a very gradual decrease in the loading force of 0.6% to 540.3 ± 1.7 N. From the loading history and with such a small force drop, it can be concluded that the specimen is highly resistant to cyclic loading while maintaining stable mechanical properties.

**Figure 8:**
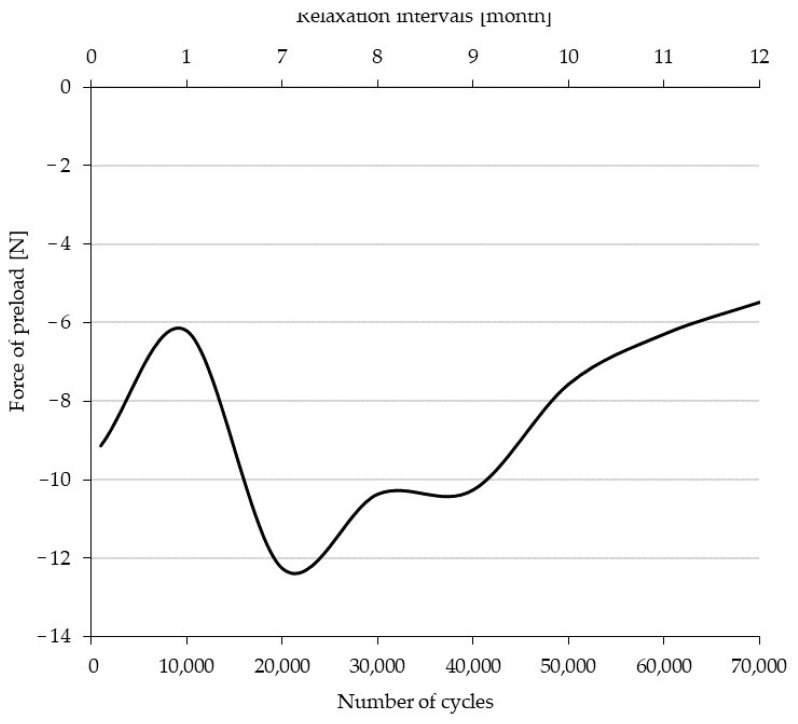
Preload force period of the ski after 0 mm deflection in the interval 0–70,000 cycles as a function of relaxation time.

The initial preload was set at 20 N. After preloading the test specimen to the desired value, the measuring device was reset. Figure 8 shows that the value after the first 1000 cycles of measurement decreased by 9.1 ± 1.1 N relative to the preset value of 20 N.

This value illustrates the reduction in the preload at the starting position of the test specimen. After 10,000 cycles, the decrease was only 6.2 ± 0.9 N, i.e., the ski is settling in the test process. After a subsequent 6-month relaxation, the force of the preload value drops by 12.2 ± 0.9 N. This step change can be observed in both Figure 7 and Figure 8, where the change in the characteristic at the 20,000th cycle can be seen. This is due, first, to the self-reinforcing process where the different phases of the ski are established in 20,000 cycles, and, secondly, to the long relaxation time of 6 months. This decrease occurs when the force of the preload has a negative effect on the flexibility of the ski, i.e., the ability of the ski to return to its initial state after a certain cyclic load. The ski achieves progressively lower decreases in the force of the preload in cycles of 20,001 to 70,000 when the relaxation time is reduced to 1 month. As the number of cycles increases, the force of the preload returns to the initial value, with a decrease of only 5.5 ± 0.8 N at 70,000 cycles.

**Figure 9:**
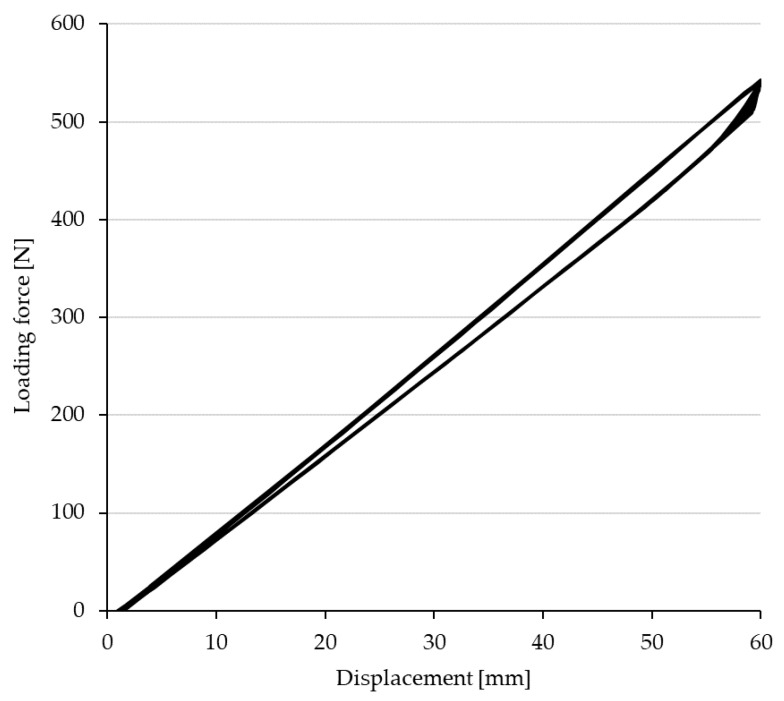
Example of part of one cycle of low cycle fatigue of a hybrid ski design.

Figure 9 shows a partial section of one low-cycle fatigue cycle of a composite hybrid ski structure.

The figure shows the progression of the test—namely, loading to maximum deflection, i.e., a 60 mm displacement of the universal testing machine crossbar, followed by a 1000× decrease in force to an initial zero crossbar displacement of the testing machine. Due to the cyclic loading, a complex state of stress developed in the ski under testing, mainly caused by this condition, and after its release, i.e., in the stress-free state, the ski tended to return to its original state. Figure 9 shows that the load and displacement loops are still the same with increasing the number of cycles. There was no tendency to change, i.e., to shift these hysteresis loops. There was no accumulation of plastic deformation manifested by displacement of these hysteretic loops. This is an important finding, given that the accumulation of plastic deformation leads to an exceedance of the elastic limit, which is manifested by delamination of the individual layers of the sandwich structure, which inevitably leads to destruction [25,26].

**Figure 10:**
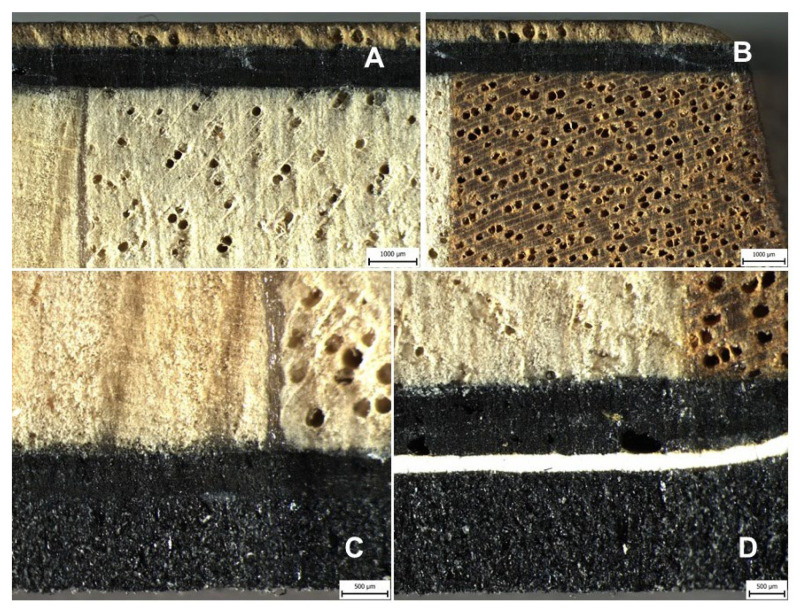
Cross-section of a hybrid sandwich ski structure after long-term low-cycle fatigue, i.e., 70,000 cycles: (**A**): top of ski—material interface wood topsheet (No. 1), E-Glass biaxial (No. 2), carbon triaxial (No. 3), carbon unidirectional (No. 4, 5, 6), E-Glass biaxial (No. 7), wood core (No. 8) (scale 1000 μm); (**B**): side of ski-material interface wood topsheet (No. 1), E-Glass biaxial (No. 2), carbon triaxial (No. 3), carbon unidirectional (No. 4, 5, 6), E-Glass biaxial (No. 7), wood sidewall (No. 9) (scale 1000 μm); (**C**): lower part of the ski—wood core (No. 8), E-Glass biaxial (No. 10), carbon triaxial (No. 11), base (No. 15) (scale 500 μm); (**D**): bottom of ski—wood core (No. 8), E-Glass biaxial (No. 10), carbon triaxial (No. 11), VDS rubber (No. 13), base (No. 15) (scale 500 μm).

**Figure 11:**
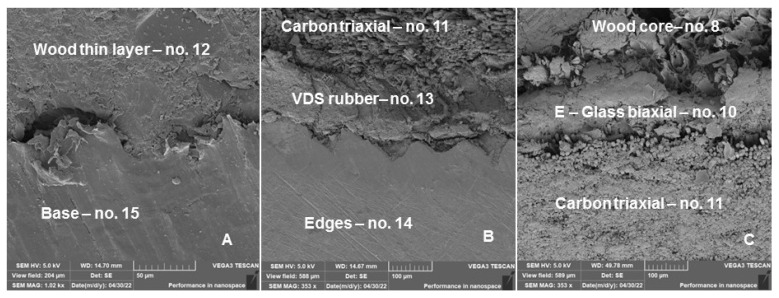
Cross-section of a hybrid sandwich ski structure after the end of long-term low-cycle fatigue, i.e., 70,000 cycles—SEM pictures: (**A**): Interaction of the base (No. 15) and wood thin layer (No. 12) interface—central part of the ski cross-section (MAG 1020×), (**B**): Interaction of the interfaces edges (No. 14), VDS rubber (No. 13) and carbon triaxial (No. 11)—extreme edge of the ski cross section (MAG 353×), (**C**): Interaction of the interfaces carbon triaxial (No. 11), E Glass biaxial (No. 10) and wood core (No. 8)—central part of the ski cross section (MAG 353×).

**Figure 12:**
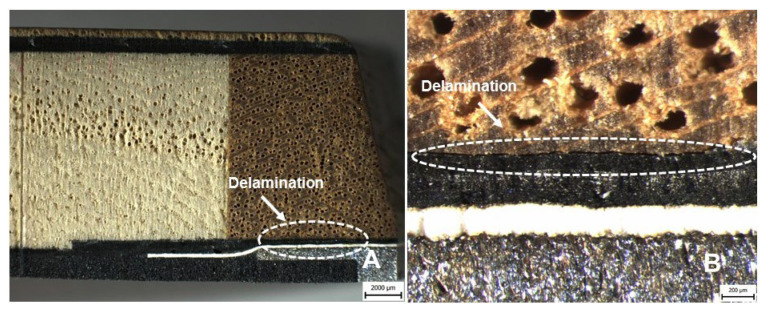
Cross-section of the hybrid sandwich ski structure after the end of long-term low-cycle fatigue, i.e., 70,000 cycles: (**A**): Macroscopic picture of delamination in the lower part of the ski at the interface of wood sidewalls (No. 9) and carbon triaxial (No. 11) (scale 2000 μm), (**B**): Microscopic picture of delamination in the lower part of the ski at the interface of wood sidewalls (No. 9) and carbon triaxial (No. 11) (scale 200 μm).

**Figure 13:**
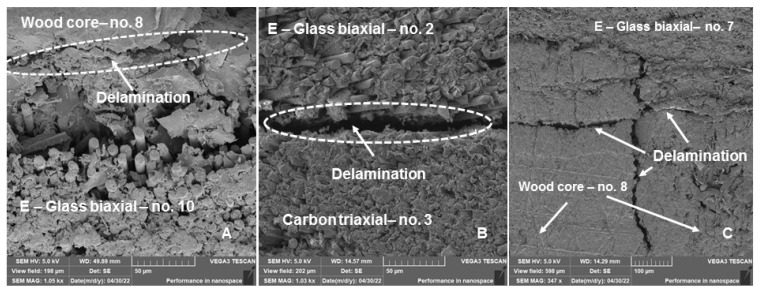
Cross-section of the hybrid sandwich ski structure after the end of long-term low-cycle fatigue, i.e., 70,000 cycles—SEM pictures: (**A**): Delamination of the wood core layer (No. 8) and E-Glass biaxial (No. 10) composite layer (MAG 1050×), (**B**): Delamination between E-Glass biaxial (No. 10) and carbon triaxial (No. 11) composite layers (MAG 1030×), (**C**): Delamination of wood core (No. 8) and its interaction with E-Glass biaxial (No. 10) (MAG 347×).

Long-term low-cycle fatigue did not affect the mechanical properties of the ski and there was no change in the shape or delamination of the individual layers of the structure. The delamination of the individual layers of the hybrid sandwich construction of the test ski after the end of the long-term low-cycle fatigue, i.e., 70,000 cycles, is evident from the cross-sections shown in Figure 10, Figure 11, Figure 12 and Figure 13. Figure 10 shows cross-sections of the hybrid sandwich ski structure, which show the different material composition described in Figure 2 and Table 2 (Layer in the ski structure; Layer position no.). Figure 10 shows the strong adhesion and cohesion bonds within the tested materials and at the interface of their bonding by adhesive.

Figure 11 shows cross-sectional images of the tested ski using SEM analysis and the transition region of the adhesive bonding of the different materials of the sandwich hybrid construction of the tested ski. A significant conclusion is that the strong adhesive bonding of the base, edges and other layers necessary for the safe use of the ski are in direct interaction with the slope. Interaction of the functional surface with the external environment is one of the basic requirements to ensure the functioning of the production [27,28]. Delamination of the base and edges was not demonstrated by SEM image analysis.

The delamination was determined in the lower part of the tested ski at the interface between the wood sidewalls (No. 9) and carbon triaxial (No. 11) materials, i.e., the composite layer, see Figure 12.

The delamination was not connected to the surface of the ski but was located about 5 mm from the outer surface. A more detailed investigation using SEM analysis detected additional areas of delamination (Figure 13). Figure 13A shows delamination at the interface of the wood core (No. 8) and the E-Glass biaxial (No. 10) composite layer, which was 38.64 ± 6.47 µm wide. Another weak point was the interface of the E-Glass biaxial (No. 2) and carbon triaxial (No. 3) composite layers. The delamination joint was 11.60 ± 1.94 µm wide (see Figure 13 B). Figure 13C also shows the delamination inside the wood core (No. 8), which was 11.48 ± 2.78 µm wide.

Figure 10 and Figure 12 were created by the stereoscopic microscope Olympus SZX10 (Olympus Czech Group, s.r.o., Prague, Czech Republic). Figure 11 and Figure 13 were created in the scanning electron microscope (SEM) Tescan VEGA 3 (Tescan Brno s.r.o., Brno, Czech Republic).

## 4. Conclusions

This paper presents the low-cycle fatigue results of a newly developed and tested sandwich hybrid ski design. The results of the research, based on long-term cyclic experimental tests comprising 70,000 cycles focused on the effect of repeated deformation of the composite hybrid ski structure, showed that the deformation did not have a significant effect on the change in the maximum force achieved during this deformation or the change in shape and dimensions, nor did it have an effect on the damage/delamination of the hybrid sandwich ski structure that would be noticeable to the normal user.

The findings presented in this paper are summarized in the following points:

- In the 20,000th cycle, the ski was stiffened at a deflection of up to 60 mm, i.e., a self-reinforcing process. This stiffening was maintained with a slight decrease to 70,000 cycles to 540.4 ± 0.8 N. Additionally, at 20,000 cycles, the largest decrease in the force of the preload at the 0 mm position relative to the set value was 12.2 ± 0.9 N, i.e., a decrease in flexibility. As the number of cycles increases, the force of the preload value returns to the baseline value, with a decrease of only 5.5 ± 0.8 N at 70,000 cycles.
- The results show that the ski retains its strength and elasticity under cyclic loading and no permanent deformation occurs, but it is important to consider the relaxation time of the ski. Longer relaxation times can affect the ski’s performance. This can be avoided by determining the storage conditions. To specify the correct conditions, the ski to be tested should be subjected to further research.
- Visual inspection of the sandwich hybrid construction of the ski revealed no visible delamination associated with the surface layers of the tested ski or change in shape. Using optical analysis, local delamination between the wood core and the composite layers E-Glass biaxial and carbon triaxial was demonstrated in the cross section. In addition, cohesive failure of the wood core was detected. This local delamination did not significantly affect the integrity of the hybrid sandwich construction of the tested ski. Another significant conclusion from the image analysis is that there was no damage to the adhesive bonds between most of the different materials used in the hybrid sandwich construction of the ski due to low-cycle fatigue. Particularly, there was no damage between the base, edges and other layers, which are necessary for the safe use of the ski and are in direct interaction with the slope.

## Figures and Tables

**Table 1:** Ski parameters.

Ski Length (cm)	Ski Width (mm)	Radius (m)	Ski Weight (g per Ski)
181	131-96-116	21	1450

**Table 2:** Composition of materials in the ski structure.

Layer in the Ski Structure	Layer Orientation	Area Weight	Layer Position No.
-	[°]	[g × m^−2^]	
Wood topsheet	-	-	1
E–Glass biaxial	0/90	100	2
Carbon triaxial	0/±45	400	3
Carbon unidirectional	0	100	4
Carbon unidirectional	0	100	5
Carbon unidirectional	0	100	6
E–Glass biaxial	0/90	100	7
Wood core	-	-	8
Wood sidewalls	-	-	9
E–Glass biaxial	0/90	100	10
Carbon triaxial	0/±45	400	11
Wood thin layer	-	-	12
VDS rubber	-	-	13
Edges	-	-	14
Base	-	-	15

**Table 3:** Test parameters and results low-cycle test of hybrid sandwich ski construction.

Number of Cycles (-)	Relaxation Intervals (Month)	Loading Force (60 mm Bending) (N)	Force of Preload (0 mm Bending) (N)
1000	0	518.1 ± 5.4	−9.1 ± 1.1
10,000	1	514.0 ± 4.2	−6.2 ± 0.9
20,000	7	543.6 ± 1.7	−12.2 ± 0.9
30,000	8	544.2 ± 1.0	−10.4 ± 1.2
40,000	9	543.7 ± 1.4	−10.2 ± 1.3
50,000	10	540.3 ± 1.7	−7.6 ± 1.3
60,000	11	541.4 ± 0.5	−6.3 ± 0.8
70,000	12	540.4 ± 0.8	−5.5 ± 0.8

## Data Availability

Not applicable.
